# Cerebral vein thrombosis after ChAdOx1 nCov‐19 vaccination: Long‐term outcome of four patients

**DOI:** 10.1002/rth2.12844

**Published:** 2022-11-16

**Authors:** Deepa J. Arachchillage, Christina Crossette‐Thambiah, Namir Asmar, Saipriya Ramji, Mike Laffan

**Affiliations:** ^1^ Centre for Haematology, Department of Immunology and Inflammation Imperial College London London UK; ^2^ Department of Haematology Imperial College Healthcare NHS Trust London UK; ^3^ Department of neuroradiology Imperial College Healthcare NHS Trust London UK

Vaccine‐induced immune thrombotic thrombocytopenia (VITT) is a rare prothrombotic syndrome associated with an adenoviral vector‐based vaccine ChAdOx1 CoV‐19 vaccine (AstraZeneca, University of Oxford) or Ad26.COV2.S vaccine (Janssen; Johnson & Johnson). VITT is characterized by thrombosis, often at unusual sites, particularly cerebral venous sinus thrombosis (CVST) with thrombocytopenia, low fibrinogen, and significantly raised D‐dimer level.^1^ A large UK‐based study reported an overall mortality rate of 22%, rising to 73% in patients with platelet count less than 30 × 10^9^/L and intracerebral hemorrhage (ICH).^2^

Early recognition and prompt treatment reduced mortality of VITT from approximately 50% in the first series reported in April 2021 to 22% by June 2021 in the United Kingdom and more recently to about 5% observed in Australia, probably due to earlier recognition and prompt treatment.^3^ The pathophysiology of VITT remains incompletely defined but appears to be an immune‐mediated process, similar to that of heparin‐induced thrombocytopenia (HIT). It is associated with the development of IgG antibodies directed against platelet factor 4 (PF4) but without exposure to heparin. Thrombosis can be venous or arterial or both and often develops at multiple sites. Laboratory markers can demonstrate a consumptive coagulopathy, with thrombocytopenia, hypofibrinogenemia, and grossly elevated D‐dimers. Notably, PF4 antibodies are most reliably detected using ELISA while chemiluminescence immunoassays assays produce false‐negative results. CVST has been reported to be the predominant thrombotic manifestation, whereas it is rare in HIT. In a recent systematic review of 23 case series of HIT including 1220 patients with HIT, only 27 had CSVT(1.6%)^4^ compared to 50% of patients with VITT (110/220).^2^ The presence of ICH is associated with high mortality in patients with CVST, reaching 73% in patients with VITT‐associated CVST.^2^ However, long‐term outcome data of the patients presenting with CVST associated with VITT are lacking.

We report a single‐center experience of beyond 1‐year (14 months) outcomes of four female patients, aged 41–46 years, who developed CVST with or without thrombosis at other sites due to VITT during May 7–24, 2021, in London, United Kingdom (Table S1). These four patients represented all patients admitted to our hospitals with VITT during this period. The institutional review board approved this study, and informed written consent was obtained from all patients. Each presented with headache and neurological deficit. Two patients had subarachnoid hemorrhage. A functional assay to demonstrate heparin‐independent platelet‐activating anti‐ PF4 antibodies without additional PF4 was performed for the diagnosis using whole‐blood impedance aggregometry in addition to anti‐PF4 ELISA. Aggregation of donor platelets after incubation with serum from the patients was measured in the presence of low (0.43 IU/ml) or high (100 IU/ml) concentrations of unfractionated heparin (UFH) and in the absence of added heparin (saline). Serum from a healthy donor and serum from a patient with confirmed classical HIT were also tested. Serum from patients with classical HIT showed marked platelet aggregation with low‐dose UFH but not with high dose UFH. Serum from all four patients with VITT showed higher (spontaneous)  platelet aggregation in the absence of heparin (with saline) compared to serum from patient with classical HIT and the healthy control. When serum from patients with VITT was incubated with low‐ or high‐dose UFH, there was a variable response in platelet aggregation.^5^ All four patients received a uniform management approach with immediate transfer to an intensive care unit with combined hyperacute stroke unit and neurosurgical presence on initial presentation. Urgent plasma exchange (PLEX) with Octaplas was initiated within 24 h and continued for 5 days. Alongside PLEX, intravenous (IV) immunoglobulin at 1 g/kg in two divided doses and high‐dose steroids (1 g IV methylprednisolone followed by 20 mg dexamethasone IV/oral for 4 days) were started irrespective of the platelet count, as the benefit of the steroids outweighs the side effects related to a short course of steroids. Anticoagulation was initiated with argatroban at presentation despite the low platelet count due to concern of the progressive CVST. Target activated partial thromboplastin time ratio was 1.5–2.0 for the first 48 h, increased to 2.0–2.5 in the absence of further bleeding or deterioration on repeat imaging in those with subarachnoid hemorrhage. There was no progression of ICH with argatroban, and platelet count gradually improved following the start of IV IgG and high‐dose steroids^5^ (Figure 1A–D). A single dose of rituximab (375 mg/m^2^) was given to two patients (Figure 1A,B). Patient 1 was given rituximab in addition to PLEX, high‐dose steroids, and IV IgG, as she presented with severe thrombocytopenia and subarachnoid hemorrhage, with a history of being semiconscious for 96 h with neurological features and laboratory features associated with poor outcome. The second patient received rituximab since she continued to drop her fibrinogen level and developed further thrombotic events despite anticoagulation with argatroban, plasma exchange, and high‐dose steroids.

**FIGURE 1:**
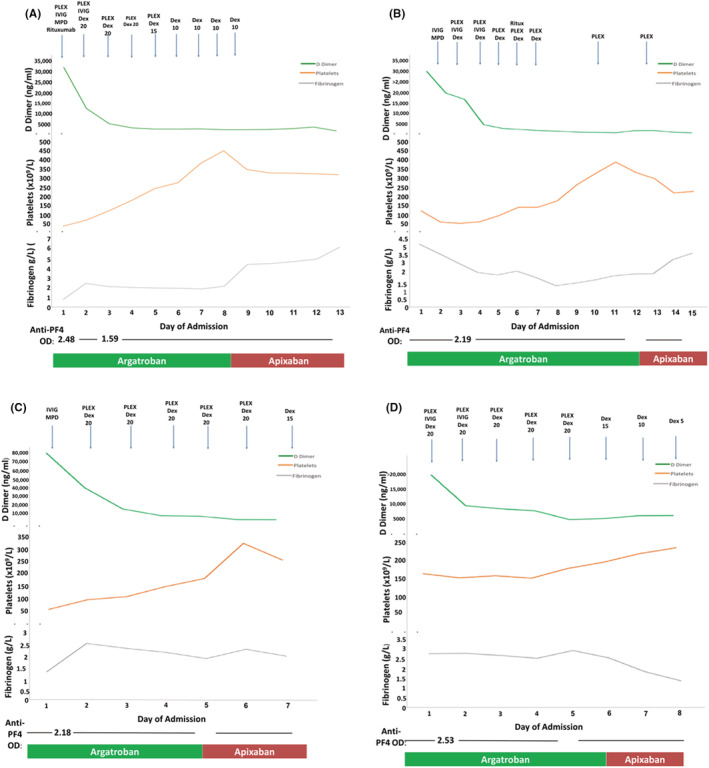
Acute treatment and changes of hematological parameters over the days since admission to discharge from hospital—Patients 1–4. (A) Patient 1; (B) Patient 2; (C) Patient 3; (D) Patient 4. anti‐PF4, anti–platelet factor 4; DEX 20, dexamethasone 20 mg; DEX 15, dexamethasone 15 mg; DEX 5, dexamethasone 5 mg; DEX10, dexamethasone 10 mg; IVIg, intravenous immunoglobulin; MPD, methylprednisolone; OD, optical density; PLEX, plasma exchange.

All patients survived with complete clinical and laboratory resolution and were discharged on apixaban 5 mg bd. Vigilant follow‐up comprised twice weekly platelet count and anti‐PF4 antibodies in the first 6 weeks and less frequently for 6 months. Despite normalization of platelet count and coagulation markers (including fibrinogen and D‐dimer), all four patients complained of frequent headache and high levels of anxiety.

There was no demonstrable antispike antibody response following the ChAdOx1 nCoV‐19 vaccine, although two had received rituximab at least 10 days following vaccination. However, all subsequently showed a good response following mRNA BNT162b2 (Pfizer–BioNTech) vaccination 12–16 weeks after the diagnosis of VITT (Table S2). Clinically symptomatic thrombosis or increase in anti‐PF4–heparin IgG ELISA did not occur in any patient (Figure 2).

**FIGURE 2:**
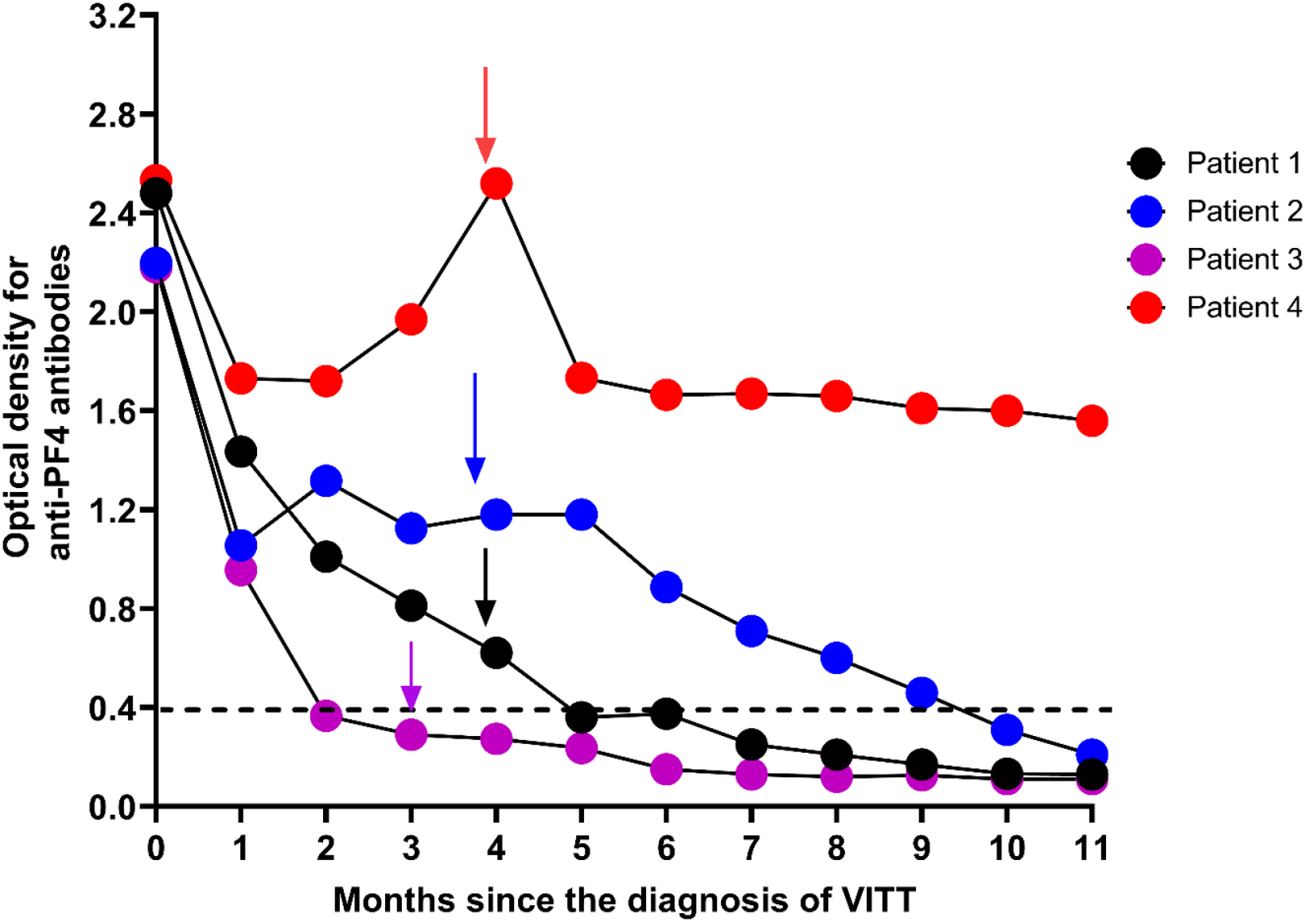
Anti‐PF4 antibodies in four patients with VITT over the follow‐up period. Arrows indicate the time of Pfizer–BioNTech vaccine administration for each patient. Black dotted line indicates the cutoff optical density value (>0.4) considered to be positive for anti‐PF4 antibody ELISA assay for VITT antibodies. anti‐PF4, anti–platelet factor 4; VITT, vaccine‐induced immune thrombotic thrombocytopenia

This is in keeping with other reported studies^6^, ^7^ including 40 patients, of which 26 had confirmed VITT and others had probable (2 patients) or possible (12 patients) VITT.^7^ Of these 40 patients, the majority (33/40) received BNT162b2 (Pfizer–BioNTech) and the others ChAdOx1 nCoV‐19 (5/40) or mRNA‐1273 (Moderna) (2/40) as their second dose. None of these patients had evidence of relapse.

Repeat brain magnetic resonance imaging (MRI) and magnetic resonance venogram (MRV) at 4 months demonstrated resolution of CVST in all patients except Patient 1, whose MRV showed a 3‐mm maximum intensity projection (MIP) at the level of the right jugular bulbs (Figure 3). The MRV repeated at 10 months remained significantly unchanged (Figure 3C).

**FIGURE 3:**
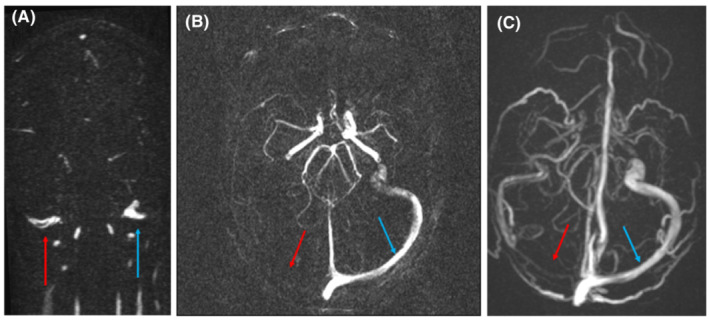
Magnetic resonance venography (MRV) of Patient 1 with 3‐mm maximum intensity projection (MIP) at the level of the jugular bulbs (A) and MRV of the transverse sinus at 4 months (A and B) and 10 months following the diagnosis of VITT. (A) MRV 29/08/21. 3 mm maximum intensity projection (MIP) at the level of the jugular bulbs. Filling defect can be seen within the right jugular bulb in keeping with ongoing thrombus (red arrow). The left jugular bulb (blue arrow) shows no filling defect. (B) MRV reconstructed image (MRV 29/08/2). The left transverse sinus (blue arrow) demonstrates normal flow; however, there is no/slow flow within the right transverse sinus (red arrow) likely secondary to the distal obstruction, rather than due to extending thrombus. (C) MRV reconstructed image on 03.04.22. The left transverse sinus (blue arrow) demonstrates normal flow, however, there is no/slow flow within the right transverse sinus (red arrow) likely secondary to the distal obstruction, rather than due to extending thrombus. Appearances are not significantly changed from 29.08.22.

Lung perfusion single photon emission computed tomography (CT), CT of the abdomen and Doppler ultrasound showed complete resolution in those with pulmonary embolism, portal vein thrombus (Figure S2), and deep vein thrombosis, respectively.

By 10 months from the diagnosis of VITT, anti‐PF4 antibodies were normalized in three patients (Patients 1–3), but Patient‐4 retained detectable antibody greater than 0.4 optical density (Figure 1). At 10‐month follow‐up, factor VIII, von Willebrand factor antigen (VWF), and VWF ristocetin cofactor, which were elevated at the acute presentation, had normalized except in Patient 4 (Figure S1).

Although Patient 4 had detectable anti‐PF antibodies, their serum failed to activate donor platelets in the presence or absence of heparin the same as all patients. At 14‐month follow‐up, platelet count, D‐dimer, and fibrinogen levels remained normal in all patients. Two patients received anticoagulation with apixaban 5 mg bd until anti‐PF4 antibodies normalized, and the patient who had residual CVST remained on anticoagulation for 12 months. The patient who had detectable anti‐PF4 antibodies at 10 months, developed abnormal liver function tests (raised bilirubin and serum alanine transferase) at 4 months following the start of apixaban 5 mg bd and switched to fondaparinux 7.5 mg daily and remained on this for 12 months.

Cerebral venous sinus thrombosis is a relatively rare and unusual site of venous thromboembolism (VTE), with estimated incidence around 2.85–3.16 cases per 100,000 of hospitalized patients per year, which represents a distinct cause of stroke primarily affecting young adults.^8^ Factors that can contribute CVST in general are multiple, including those associated with VTE and specific local causes such as local infections, trauma, and brain tumors.^9^ Overall death and dependency rate following CVST is around 15%.^10^ However, presence of ICH is generally associated with a high mortality in patients with CVST. Around 40%–60% of patients with CVST can have evidence of ICH including parenchymal/subdural hematomas and subarachnoid hemorrhages.^11^ The frequency of ICH was around 36% (40/110) in patients with CVST associated with VITT, and mortality was (73%).^2^

Evidence on management of CVST in general is limited, and initial anticoagulation with either UFH or low‐molecular‐weight heparin is currently recommended in patients without VITT, irrespective of the presence of ICH.^10^ Nonheparin parenteral anticoagulation such as argatroban was used for patients with VITT, especially those presenting with ICH. However, there was no significant difference in the mortality among patients who received heparin following VITT (20%) compared to patients who received nonheparin anticoagulants (16%),^2^ suggesting that heparin may be used in patients with VITT. Around 5% of patients may have antibodies that cross‐react with PF4–heparin complexes.^3^

The resolution of CVST outside the context of VITT has been reported in several studies in follow‐up imaging. In a study of 33 patients with CVST, recanalization occurred in most patients (29 [87.9%]), of which only 6 (21%) had complete resolution of the thrombosis.^12^ However, the author reported that the clinical outcome was not related to recanalization. In another study of 53 patients with CVST, follow‐up MRI was performed in only 15 patients, showing partial or complete recanalization of thrombosis in 14 cases.^13^ In a systematic review and meta‐analysis to assess the recanalization in CVST, which included 19 studies in the final analysis, 694 patients had recanalization in 818 patients with CVST accounting for the overall pooled proportion of patients achieving recanalization was 85% (95% confidence interval [CI], 80–89). When only studies with higher methodological quality were included in the analysis, the recanalization rate was 77% (95% CI, 70–82). Patients with recanalization had significantly better outcome when assessed with modified Rankin Scale (0–1).^14^

The main limitation of this study is that it is based on only four patients. However, the study reports an excellent outcome in all four patients.

The four cases of VITT presenting with CSVT in this case series represented an excellent outcome likely attributable to an aggressive treatment approach with multidisciplinary team involvement from the outset.

## AUTHOR CONTRIBUTIONS

DJA designed the study, collected and interpreted the data, drew some of the figures, and wrote and reviewed the manuscript. CCT collected the data, drew some of the figures, and wrote the manuscript. NA and SR collected the data and prepared the radiological figures. ML collected the data and reviewed the manuscript. All authors reviewed and approved the final version of the manuscript.

## FUNDING INFORMATION

DJA is funded by MRC UK (MR/V037633/1).

## RELATIONSHIP DISCLOSURE

DJA received research funding from Bayer plc and Leo Pharma (not related to this study). ML received consultation and speaker fees from Astra‐Zeneca, Sobi, Leo‐Pharma, Takeda, and Pfizer. The remaining authors have no conflicts of interest to declare.

## Supporting information


Appendix S1

Appendix S2
