# Supplementary material for: Cerebral vein thrombosis after ChAdOx1 nCov‐19 vaccination: Long‐term outcome of four patients
Source: Res Pract Thromb Haemost. 2022 Nov 16;6(8):e12844. doi: 10.1002/rth2.12844 (PMC9667410; doi:10.1002/rth2.12844)
Supplement: Supplementary file 1 — Appendix S1 [file RTH2-6-e12844-s002.docx]

**Appendix:** **Long-term outcome of cerebral venous sinus thrombosis following ChAdOx1 nCov-19 Vaccination: Single centre experience**

**Table of contents page**

**Table S1: Characteristics of the four patients with VITT at presentation…………………………….2**

**Figure S1 A: Factor VIII of the four patients with VITT over time……………………………………….4**

**Figure S1 B: Von Willebrand factor antigen (VWF Ag) of the four patients with VITT over time……………………………………………………………………………………………………………………………… 5**

**Table S2: Antibodies against the vaccination following first dose of ChAdOx1 nCoV-19 in May 2021 and 12-16 weeks following the Pfizer–BioNTech vaccine………………………………….6**

**Figure S2: CT abdomen of the patient 4 at the presentation with the diagnosis of VITT and 3 months following the diagnosis of VITT……………………………………………………………………… 7**

**Table S1: Characteristics of the four patients with VITT at presentation**(1).

**Table S1: Characteristics of the four patients with VITT at presentation**(1).

|  | **Patient 1** | **Patient 2** | **Patient 3** | **Patient 4** |
| --- | --- | --- | --- | --- |
| **Sex** | Female | Female | Female | Female |
| **Age** | 46 | 41 | 46 | 43 |
| **Past Medical History** | Migraine | Migraine | Migraine | Hypertension  DVT |
| **Clinical presentation** | headache, collapse, hemiparesis | Headaches,  Vomiting,  abdominal pain | headache | shortness of breath, collapse headache, paraesthesia |
| **No. of days post vaccine** | 14 | 9 | 9 | 28 |
| **Thrombosis** | CVST | CVST  Portal vein thrombus  PE | CVST  Hepatic vein thrombus  Portal vein thrombus | CVST  Pulmonary saddle embolus |
| **Bleeding** | Subarachnoid haemorrhage | None | None | Subarachnoid haemorrhage |
| **Platelets (150-400 x10^9^)** | 39 | 125 | 57 | 161 |
| **D Dimer (<500 ng/ml) FEU** | >20,000 | >20,000 | >20,000 | >20,000 |
| **Fibrinogen (1.9-4.3g/L)** | 0.7 | 4.26 | 1.4 | 2.75 |
| **PT (12.8-17.4 secs)** | 14.7 | 13.5 | 12.0 | 14.8 |
| **APTT (25.0-35.0secs)** | 43.2 | 28.5 | 39.0 | 26.2 |
| **HemosIL AcuStar HIT-IgG* (<1u/ml)** | 0.05 (neg) | 0.03 (neg) | 0.02 (neg) | 0.04 (neg) |
| **Immucor ELISA PF4 HIT-IgG (<0.4OD)** | 2.48 | 2.19 | 2.18 | 2.53 |
| **HYPHEN BioMed ELISA PF4 HIT-IgG (OD) (<0.4OD)** | 1.67 | 1.89 | 1.78 | 1.92 |

CVST = cerebral venous sinus thrombosis

.

.

**Figure S1 A: Factor VIII of the four patients with VITT over time since diagnosis**


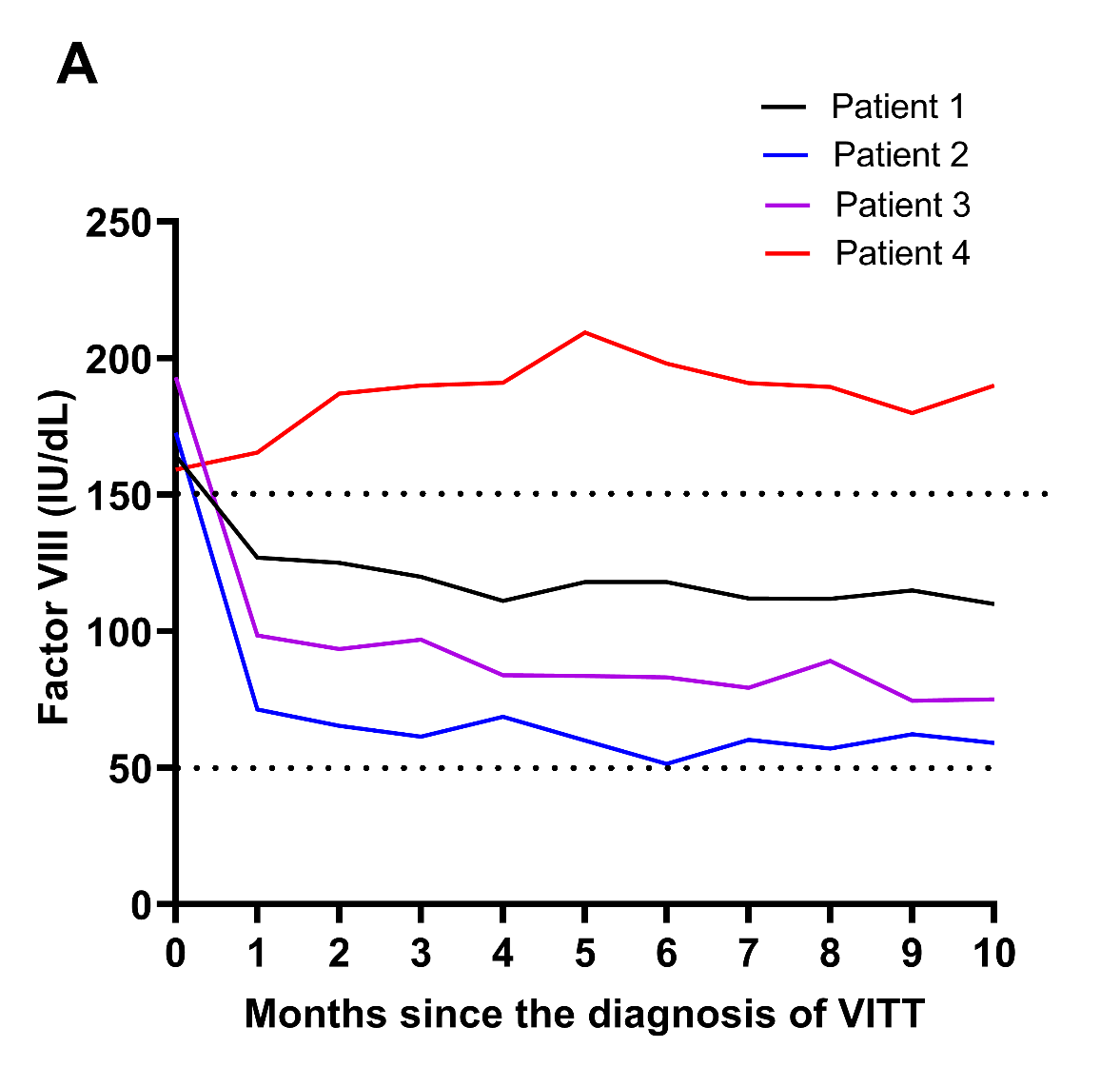


Dotted lines indicate normal reference range for factor VIII

**Figure S1 B: Von Willebrand factor antigen (VWF Ag) of the four patients with VITT over time since diagnosis**


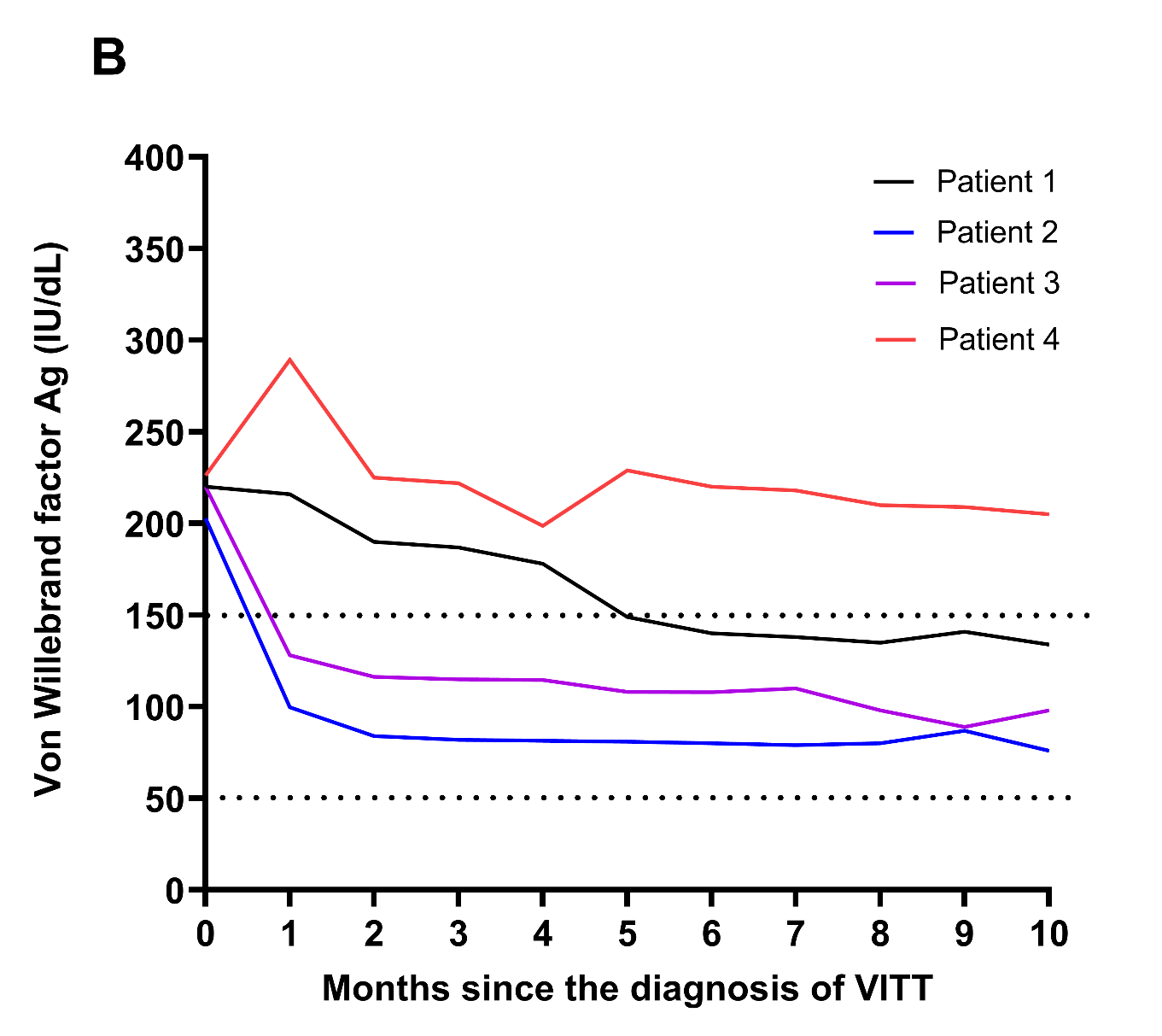


Dotted lines indicate normal reference range for Von Willebrand factor antigen

**Table S2: Antibodies against the vaccination following first dose of ChAdOx1 nCoV-19 in May 2021 and 12-16 weeks following the Pfizer–BioNTech vaccine in four patients with VITT**

|  | **Post- ChAdOx1 nCoV-19**  **S protein IgG*** | **Post-Pfizer–BioNTech vaccine**  **S protein IgG*** |
| --- | --- | --- |
| **Patient 1** | 7.16 | 249.94 |
| **Patient 2** | 17.36 | 2872.86 |
| **Patient 3** | 7.14 | 741.41 |
| **Patient 4** | 12.82 | 292.11 |

*S protein IgG levels are reported in binding antibody unit per mL (BAU/mL) based on the correlation with the WHO International Standard for anti SARS-CoV-2 antibody. The level of antibody that correlates with protection against infection, and the duration of a protective response, are currently unknown. S Protein IgG antibody assays performed using the Abbott Architect (> 7BAU/ml considered as positive)

**Figure S2: CT abdomen of the patient 4 at the presentation with the diagnosis of VITT and 3 months following the diagnosis of VITT**

**Figure S2A: 28/05/21 – portal vein thrombus (red arrow) can be seen as a filling defect originating from the portal confluence. This later resolved as shown on the figure S2B 31/08/21 (blue arrow demonstrating contrast within the portal vein).
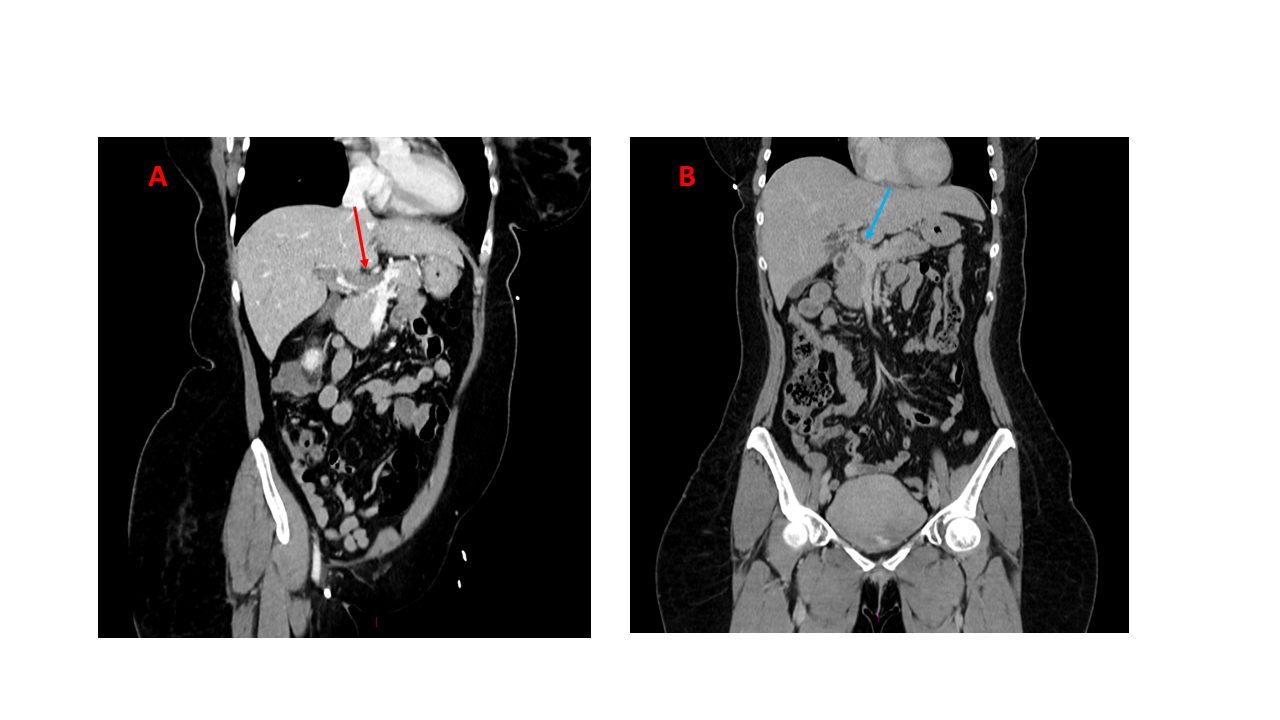
**

**References**

1. Crossette-Thambiah C, Pericleous C, Asmar N, Bomsztyk J, Ranger A, Shlebak A, et al. Clinical and biological features of cerebral venous sinus thrombosis following ChAdOx1 nCov-19 vaccination. J Neurol Neurosurg Psychiatry. 2022;93(4):445-8. Epub 2021/10/01. doi: 10.1136/jnnp-2021-327340. PubMed PMID: 34588182.
